# Dicomflex: A novel framework for efficient deployment of image analysis tools in radiological research

**DOI:** 10.1371/journal.pone.0202974

**Published:** 2018-09-11

**Authors:** Roland Stange, Nicolas Linder, Alexander Schaudinn, Thomas Kahn, Harald Busse

**Affiliations:** 1 Department of Diagnostic and Interventional Radiology, University Hospital Leipzig, Leipzig, Saxony, Germany; 2 Integrated Research and Treatment Center (IFB) Adiposity Diseases, Leipzig University Medical Center, Leipzig, Saxony, Germany; NIH Clinical Center, UNITED STATES

## Abstract

**Objective:**

Medical image processing tools in research are often developed from scratch without the use of predefined software structures, which potentially makes them less reliable and difficult to maintain. The objective here was to present and evaluate a novel framework (Dicomflex) for the deployment of tools with a uniform workflow, commonly encountered in medical image analysis.

**Materials and methods:**

The object-oriented code was developed using Matlab. Dicomflex applications follow the common workflow of image-slice selection, user interaction, image processing, result visualization and progression to next slice. The framework consists of three important classes that host functionality, two configuration files and a front end that displays images, graphs and resulting data.

**Results:**

So far, three different research tools have been created under the new framework. In comparison with previous Matlab analysis tools used at our institution, users of Dicomflex tools subjectively considered the learning phase to be shorter and handling to be simpler and more intuitive. They also highlighted the benefit and comfort of the standardized interface and predefined workflow. The framework-inherent handling of software versions was considered highly beneficial for maintenance as well as data and software management at different project stages. The clear separation of framework-related and unrelated code allows for a fast and more direct design of new tools in well-defined steps. The flexibility of the framework translates to a wide range of image processing tasks, such as segmentation, region-of-interest (ROI) analyses or computation of functional parameter maps, but is limited to 2D datasets.

**Conclusion:**

Potential medical applications include the assessment of cardiac performance, detection of cerebrovascular disease or characterization of cancerous lesions. Dicomflex tools share a similar workflow and host the pertinent functions only. This may be relevant for many image processing needs in radiological research, where quick software deployment and reliability of results is essential.

## Introduction

Review and interpretation of medical images play a key role for radiological diagnostics and procedural decisions. Over the last years, powerful computer workstations and dedicated software modules have sped up and improved these tasks. Fast and even online processing have become a reality and many processing functions, originally developed in research settings, have found their way into commercial products. For the translation of such applications, which may take many years, however, there is a continuous need to assist researchers with the quick and effective deployment of software tools for interactive processing, visualization or analysis.

Software that allows for flexible 2D or 3D visualization of medical images with a wide array of processing functions is readily available, such as MeVisLab[1], 3D Slicer[2], Osirix[3], ITK-SNAP[4], MIPAV[5], ImageJ[6], ClearCanvas (Synaptive Medical, Toronto, Canada), SliceOmatic (Tomovision, Montreal, Canada), BioImaging Suite[7] or Segment[8]. Professional coding typically ensures that programs run reliably and fast. Special interfaces for scripts and program libraries[9–12] provide options for customization.

With developer environments like Matlab (Mathworks, Natick, MA, USA), Python (Python Software Foundation, Wilmington, DE, USA), IDL (Exelis VIS, Boulder, CO, USA) or Eclipse (Eclipse Foundation, Ottawa, ON, Canada), highly customized end-user applications may be created. In medical research, tasks are usually addressed by writing small, dedicated programs. This is confirmed by our previous experience with a number of processing tools for the analysis of MRI and CT data of an institutional research cluster (Integrated Research and Treatment Center for adiposity diseases at Leipzig University Hospital, Leipzig, Germany)[13–15].

Many of these programs exhibit a relatively simple workflow of the following type: slice selection, user interaction, image processing, result visualization and progression to next slice. This justifies the design of a software template that minimizes efforts in software programming and maintenance. Unlike other user-friendly and robust Matlab UI frameworks for image analysis, for example, imagineStudios (https://github.com/imagineStudios), which effectively serve a broader scope of processing functions, the novel object-oriented framework (Dicomflex) presented here goes beyond the user interface and was deliberately designed for more routine analyses of 2D image sets and is internally optimized for the above workflow. Its applications feature the pertinent functions only and be easy to use and robust. The key classes and their functional interplay are described. A sample use case is given to evaluate the specific benefits and limitations of such a framework and to demonstrate the ease of implementation.

## Materials and methods

The framework (Dicomflex) was developed under Matlab (Version 9.1) with predefined methods from three toolboxes, namely Image Processing, Signal Processing, Statistics and Machine Learning. The Spreadsheet Link extension is generally not required but was included in the use case (Fatquant) to write the resulting data in standard spreadsheet format (Microsoft Excel). The entire framework consists of object-oriented methods that use functions provided by the Matlab environment. A readily available, open-source toolbox was used to create and access text-based configuration files, formatted in JavaScript Object Notation (JSON).

**Fig 1:**
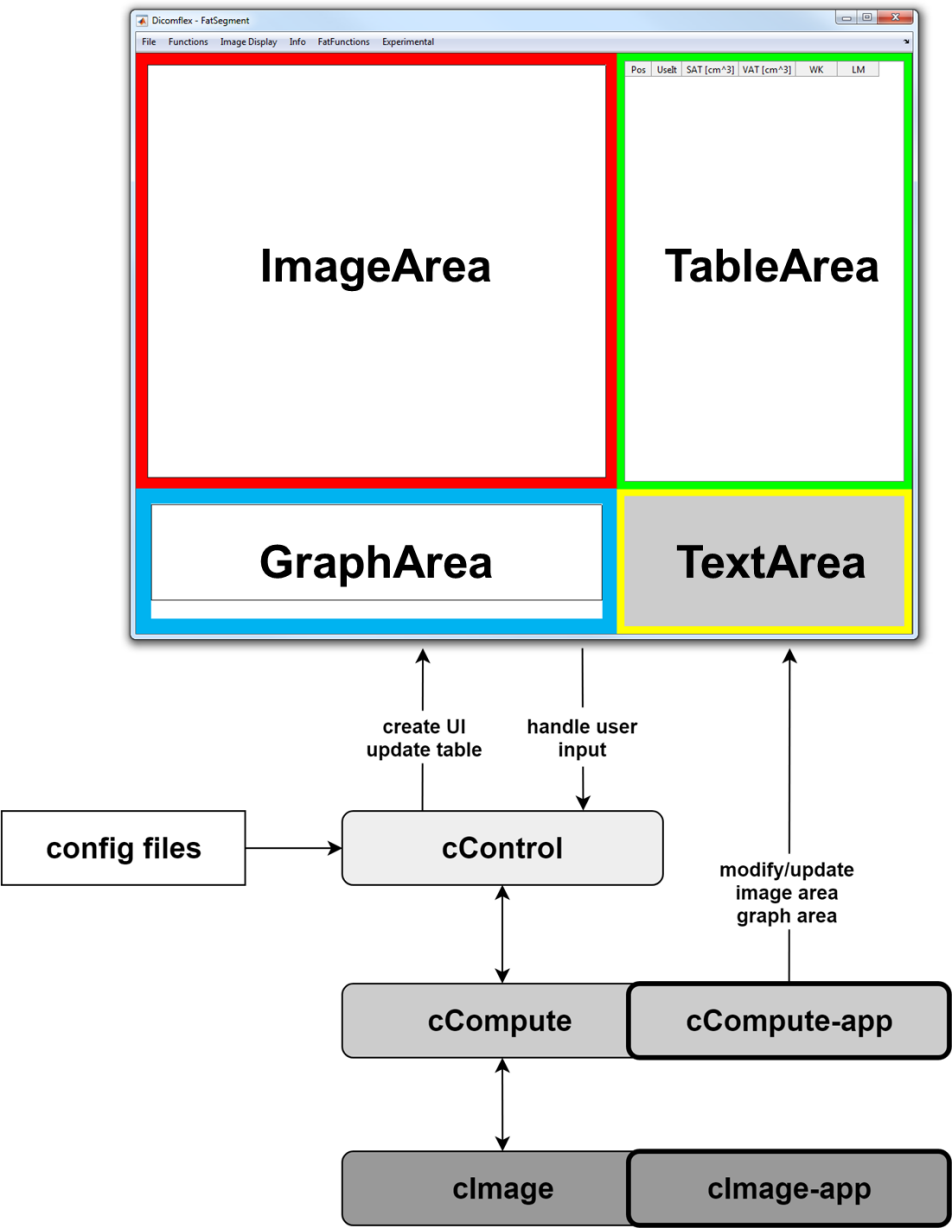
Architecture and user interface of the Dicomflex framework. The cControl class is creating a user interface at program start and controls overall program workflow. The cCompute classes are hosting computational methods whereas cImage classes store image data and provide access to basic image functions. Two configuration files store values concerning front-end appearance and program workflow. Application-specific elements are highlighted with bold frames. Classes, methods and properties are denoted by corresponding prefixes c, m and p.

The framework itself consists of three different class types and two configuration files. The underlying layout is shown in Fig 1. The adjustable front end presents relevant images, graphs and resulting data. Throughout this text, single letter prefixes c, m and p will be used for all software elements to clearly denote classes, methods and properties, respectively. Image processing and additional application-specific functions are hosted in the cCompute-app class. The interplay between user interactions and data processing is defined in the cControl class. A class group called cImage is used to store and handle image data.

### Graphical User Interface (UI)

The graphical interface between user and software structure is initialized by the cControl class and includes the following elements:

- area for image display and interactions
- area for plots and graphs
- table area with one row for each slice (and potentially multiple images)
- extendable menu bar

These elements are accessible via handles that are stored in the cControl object. All UI elements can be modified in appearance, such as size, position or color, and switched on or off by changing the associated entries in applicationConfig.

### cControl class

**Fig 2:**
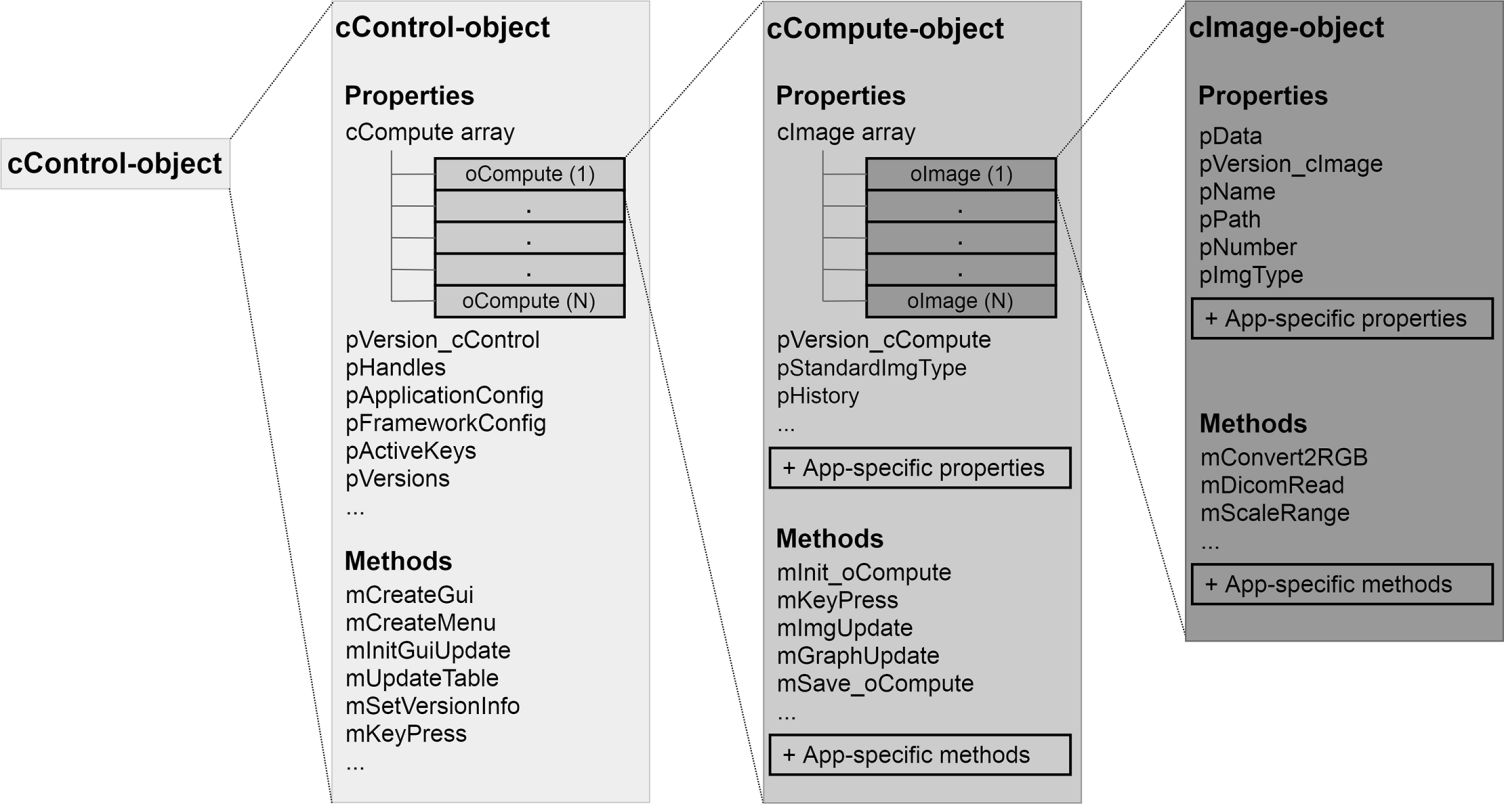
Object structure of cControl. This central object hosts an array of cCompute objects as well as methods and properties for general program control. Each cCompute object includes all properties and methods relevant to data processing. A cImage object stores a single image with its properties and fundamental methods. For a list of all methods and properties see the Git software repository (https://github.com/Stangeroll/Dicomflex).

Creation of a cControl object initializes the UI. The class cControl responds to events and directs them to the correct classes and methods. During loading of a dataset cCompute objects are created and their methods are accessible by the cControl class. The structure of a cControl object is visualized in Fig 2.

The core capabilities of cControl are the organization and connection of the cCompute class, UI and configuration files. The main events to be handled are button down in the graph and image areas, mouse wheel, key press, key release, table-cell select, table-cell edit and menu-bar click. Also, a UI-update routine is processed from cControl.

### cCompute classes

A single cCompute object contains all input and derived data for either a single image or an image set, for example, real and imaginary images of an MRI slice. The class cCompute also hosts methods for data loading and saving (mGetImgPathes, mInit_oCompute, mUpdate_cControlVersionInfo) and generic methods for updating the UI (mImageUpdate, mGraphUpdate, mTextUpdate). It is the parent class of the cCompute-app, which includes the main customized code for an application. A cCompute-app class includes mandatory methods called by cControl. Those are callbacks from user events and necessary methods for the UI-update routine (mGetImg2Display, mPostPlot, mDrawGraph, mGetTextBoxLines) as well as the mInit_oCompApp method used for application-specific data loading. In addition to these essentials, cCompute-apps may have user-defined methods. This provides the means for customization of image processing, event handling, UI-update or front-end extensions.

### cImage classes

Dicomflex has its own image class. Properties include image data, filename, path, date, a sequential number and the class version. A property pImgType is defined as a string variable and used to further identify the type of medical image, such as "T1 weighted" in the case of MR images. This information is available in cCompute to control the processing of images. The cImage class also provides image-processing functions frequently needed within Dicomflex, for example, image conversion from grayscale to RGB color space (conv2RGB) or scaling the range of pixel values (scale2). In the cImage property pData, pixel values are stored in the data type of the image to be loaded. The framework is not limited to DICOM processing but will work with image data in any format supported by MATLAB's imread function, such as, bmp, png, jpg, gif or tiff, too. Additional methods for reading and modifying individual DICOM tags have been implemented here because DICOM is the prevalent format in medical imaging.

### Configuration files

Two configuration files (JSON format) are part of Dicomflex that define the appearance of the user interface, program parameters, callbacks, file and directory names or keyboard shortcuts. While the dedicated JSON editor displays the content more clearly, configuration files can also be accessed and modified with any text editor. The file frameworkConfig.json stores general values related to UI appearance and program parameters like auto-save time or selected callbacks. Application-specific entries are stored in the file applicationConfig.json. This file includes the name tags of the table columns and their cCompute properties or methods returning the relevant data.

## Results

A framework to easily create end-user applications for the processing of medical images has been designed and applied in a use case. Software structure as well as a customizable user interface are provided and can be extended by simply modifying application-specific elements only (Fig 2). Functionality is given by the interplay of framework elements and will be reported in the following.

### User interface and basic procedures

The UI is initialized by the predefined cControl class without the cCompute and cImage class groups. UI customization is indirect and involves the configuration files frameworkConfig and applicationConfig only. It includes size adjustments, toggling of UI elements, table-header entries, program parameters as well as additional menu entries with their associated callback functions. However, it is still possible to change UI elements properties via their handle.

**Fig 3:**
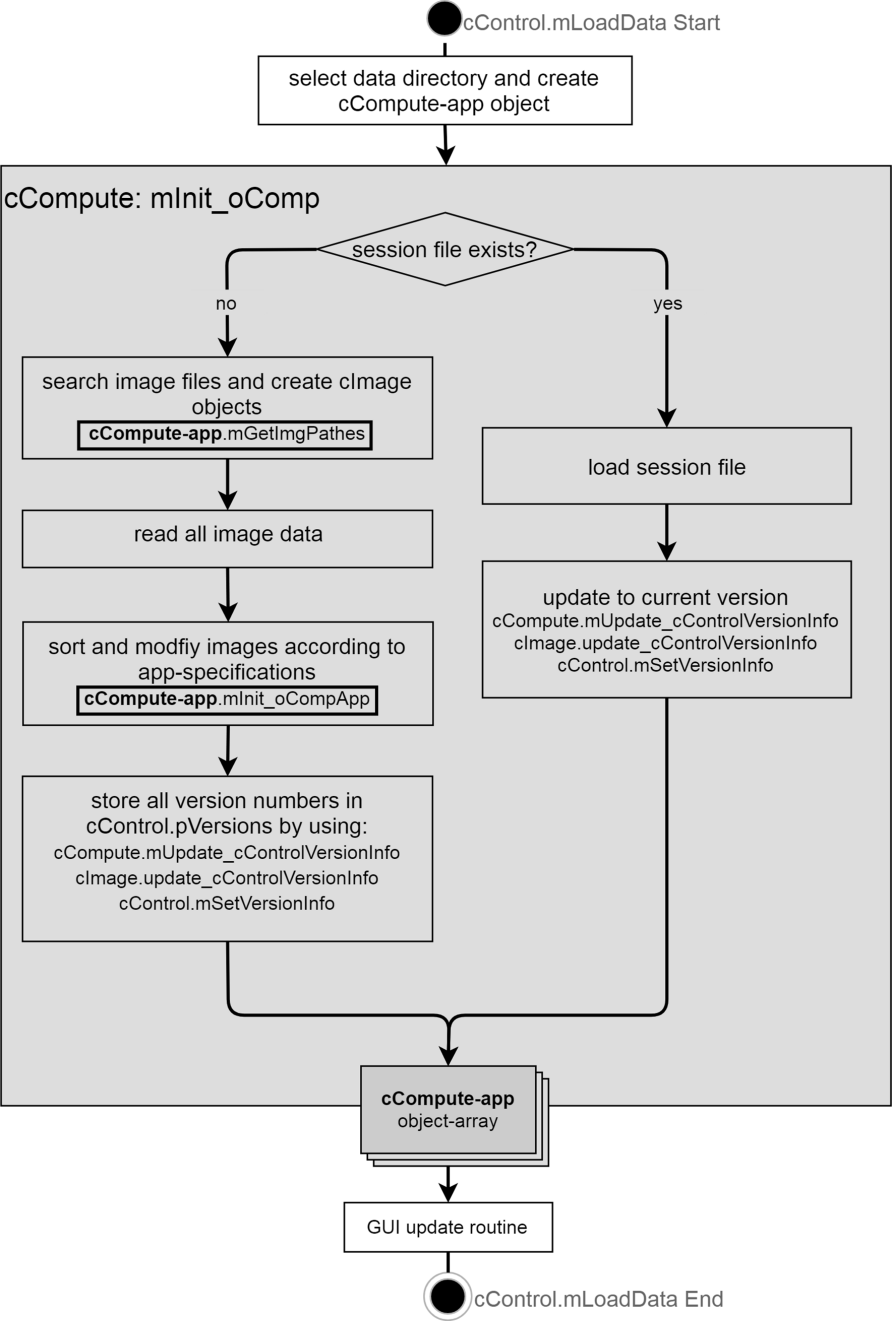
Flow chart of the mLoadData method. Within the class cControl, data are loaded via the cCompute method mInit_oCompute. In the case of a new session, mGetImgPathes determines the images according to the directory and filename pattern specified in applicationConfig (entries imgSearchDir and imgSearchName). After loading all images to cImage objects, they get merged in cCompute-app objects and filled with application-specific values in the mInit_oCompApp method. Each object corresponds to one slice with the entire oCompute array being stored in the cControl object.

The "Load Data" button event is handled by the cControl method mLoadData. After proper folder selection, cControl initializes one cCompute object of the desired cCompute-app class by reading the cComputeFcn value from applicationConfig and executing it. This gives cControl access to all application-specific properties and methods of the cCompute(-app) class, by which a cCompute object is populated with data for each table row. Fig 3 provides a flowchart of mLoadData.

Similar to the load process, the save process starts with the cControl method mSaveData. This method is executed irrespective of the application and saves the complete cCompute object array with version information to the source folder as Matlab file with the name-format *patientName_aquisitionTime_currentTime_application_data*.*mat* generated by cControl.mGetSaveFilePrefix. The mCustomSaveData method of cCompute-app is then called for any application-specific saving. This enables export of the results in a specific format (such as csv or xls), creation of processed image files or other relevant tasks.

### Version handling and backward compatibility

Backward compatibility was achieved by introducing version numbers for relevant parts, namely cControl, each cCompute class, each cImage class and both configuration files. A stored dataset includes all version numbers as well as the complete cCompute array with all images and properties. Data are released from the class definition before storage to avoid any version issues. As software undergoes development, an old dataset might not be compatible with the newest cCompute version. Therefore, all old entries get merged in a new cCompute object-array during loading. Each class has its own update function which executes the specific code to be implemented for the respective version. Version control is started after loading a dataset from the file system in the mInit_oComp method of cCompute. The most specific class or subclass of a group starts with the update and automatically passes to the next parent class until the superclass is reached.

### Event handling

**Fig 4:**
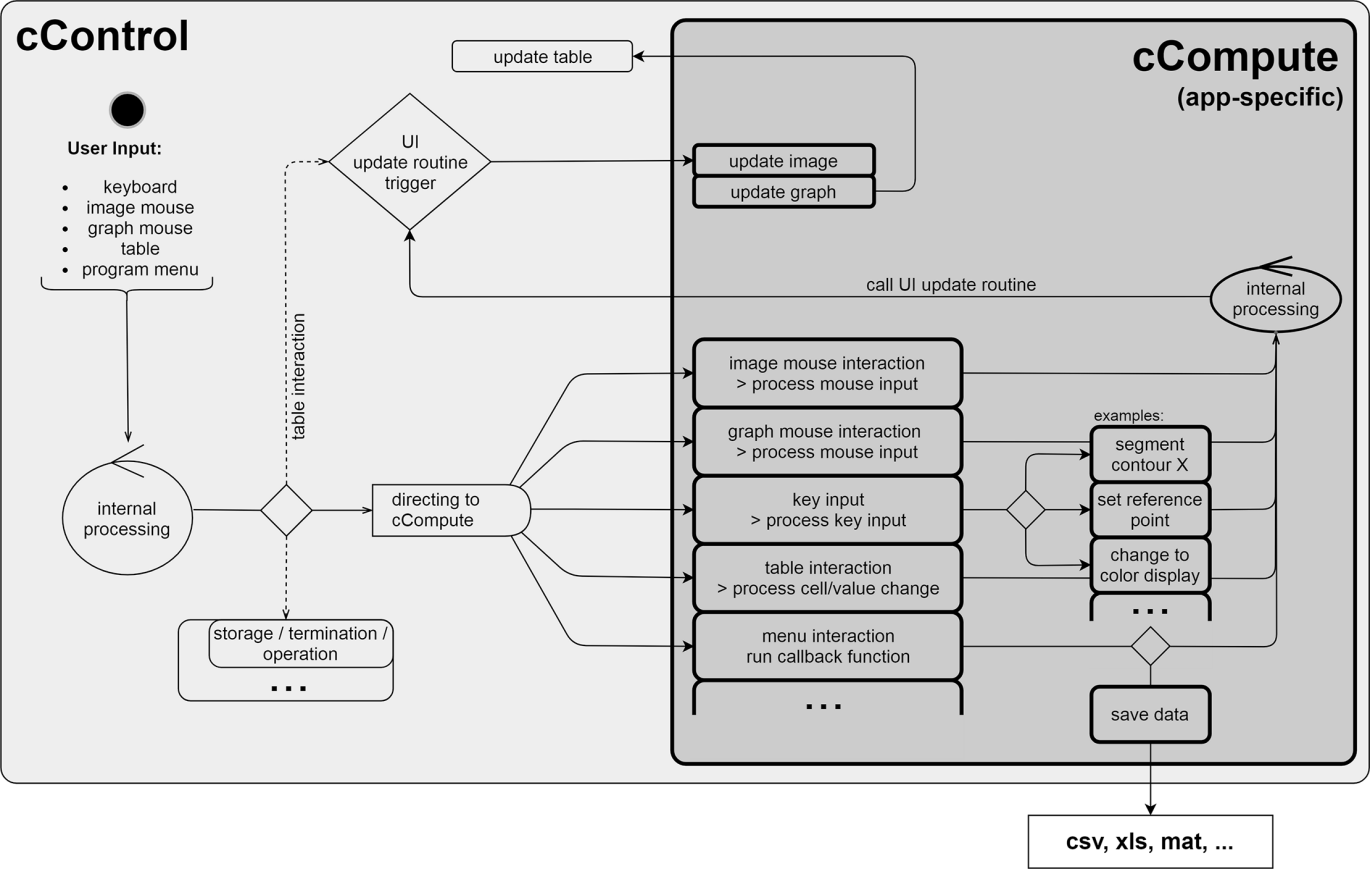
Handling of user events. A user event is registered by the cControl class, processed and, if needed, forwarded to the cCompute class, in which the event undergoes application-specific processing. Lastly, the UI-update routine is called.

User inputs are initially handled by the cControl class and processed or redirected to the cCompute or cCompute-app class, where event processing always requires a corresponding method to be called. A click on a table cell, for example, will trigger a callback to the cControl method mTableCellSelect. This method invokes the UI-update routine (part of cControl) to display information of the selected slice on the front end (Fig 4). At first, the mUpdateImage, mUpdateGraph and mUpdateText methods are triggered and the cControl object is available in those for modification of the UI. The mUpdateTable (part of cControl) is executed at last to ensure that changes to the cControl object appear in the table. The method uses information of applicationConfig and populates the table with corresponding data from the cCompute object array. The cControl class conducts the UI-update routine. It is routinely triggered after loading a dataset, selecting a different table row or clicking an application-specific menu entry. It may also be triggered from the cCompute-app class if the cControl object is available in the method.

Essential menu bar entries like "Load Data" or "Save Data" are routinely part of the UI. Specific functions can be added in applicationConfig by defining menu paths as simple string arrays and associated callback methods as strings. According to these arrays, the menu is then generated at program start by the cControl method mCreateMenu. These callbacks may take full advantage of Matlab interpretations, such as:

$$\begin{array}{c} menu(end+1).path=\{\prime Functions\prime \prime Crop\;Image\prime \};\\ menu(end).callback=\prime @(varargin)cControl.mMenuClick(@(cCompute)cControl.cCompute.mCropImage(cControl))\prime ; \end{array}$$

This example will create an additional entry "Crop Image" in the "Functions" field of the menu bar. While the callback points to the cControl method mMenuClick, the transferred function call directs to the cCompute method mCropImage, in which the cControl object is available as a transferred variable.

### Requirements and design of a use case

Execution and workflow of Dicomflex are presented for a practical implementation named Fatquant. This example was chosen because of the increasing need to quantify fat content in abdominal regions as the liver and correlate these measures with metabolic parameters or clinical findings.

Besides other techniques, multi-echo Dixon MR imaging with subsequent derivation of the so-called proton density fat fraction (PDFF) has found considerable application[16–18]. Signal intensities at different echo times (TE) show a characteristic variation caused by slight differences in the resonance frequencies of water and fat protons. Fat fraction may be derived by fitting the signal intensity from multiple acquisitions, at different echo times TE, to a mathematical model function.

**Fig 5:**
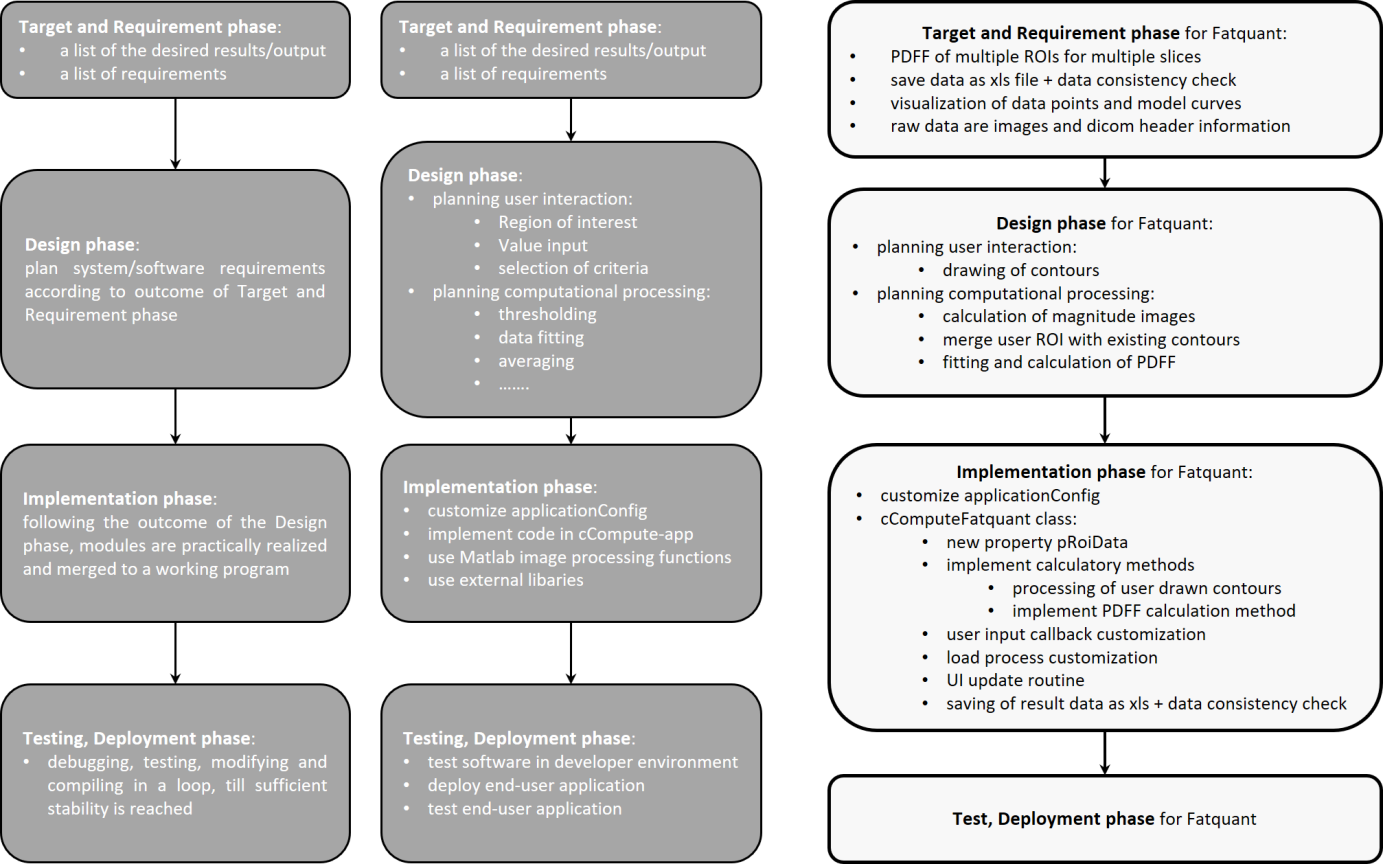
Progress of software design according to simple waterfall model. Left column: general phases; mid column: phases modified for Dicomflex implementations; right column: distinct phases of Fatquant implementation.

The fundamental work steps of framework customization are presented according to the phases defined in the so-called waterfall model[19] (Fig 5). The first step towards a custom application is the definition of input and output data rather than the particular software architecture. In Fatquant, the output is a spreadsheet file with abdominal slice positions as rows and mean fat fractions of user-defined regions of interest (ROI) as columns. The required input data consists of images at three or more echo times per slice and relevant image parameters provided in the DICOM file header.

The majority of diagnostic readings rely on magnitude MR images only. Additional phase information or both real and imaginary MR images, however, are required for proper processing of water-fat (chemical shift) images. Another parameter is the magnetic field strength, mainly 1.5 or 3 T, which defines the resonance frequencies of the fat and water protons. This value is also available in the DICOM header. Data points and model curves are plotted together to visually inspect the goodness of fit. The resulting data are verified with respect to plausibility and completeness and then written to the file system in spreadsheet format.

The drawing of three ROIs per slice is the only application-specific user interaction of Fatquant. As cControl is forwarding the mouse events in the image area to the cComputeFatquant class, processing of user-defined contours is realized in the mandatory method mImageDisplayButtonUp. The following functions are performed to determine the fat fraction in segmented ROIs:

- calculation of magnitude images (mGetMagImages)
- merging of user drawn contours with existing ROIs (mMergeContours)
- fitting of pixel intensities (mFitIntensities)
- numerical computation of fat fraction (mGetPDFF)

These functions are not described in more detail here because the focus lies on the capabilities of the framework and not on specific solutions for MRI-based fat quantification.

### Implementation and deployment of a use case

Implementation starts with the applicationConfig file, which is created by executing the customized configFileCreator.m code, in which all entries are clearly listed for editing. Table 1 shows an overview of relevant entries for the Fatquant use case.

**Table 1:** Relevant entries in the configuration file of the Fatquant application.

Parameter / Entry	Value	Use
applicationName	'Fatquant'	loading or saving of datasets
ComputeClassFunctionCall	'@cComputeFatquant'	loading of datasets
saveDataFunction	'@mSaveXLS'	saving of datasets
imageClassFunctionCall	'@cImageDcm'	loading of datasets
imageNames	{'Real', 'Imaginary'}	loading of datasets
imageDirectoryPattern	{'Dixon*', 'Dixon*'}	loading of datasets
imageFilenamePattern	{'*_IM_*.dcm', '*_RE_*.dcm'}	loading of datasets
image display visibility	'on'	UI creation
graph display visibility	'on'	UI creation
table visibility	'on'	UI creation
table column headers	{'Pos', 'Done', 'PDFF(1)', ' PDFF(2)', 'PDFF(3)'}	UI-update routine
table entry calls	{'pSliceLocation', 'pSliceDone', 'mGetPDFF(1)', 'mGetPDFF(2)', 'mGetPDFF(3)'}	UI-update routine
menu entries	{'Functions' 'Copy ROIs'} {'Functions' 'Paste ROIs'} {'Image' 'Show Magnitude'} {'Image' 'Show RealPart'} {'Image' 'Show ImaginaryPart'}	customization of file menu
menu entry callbacks	'@(varargin)d.mMenuCallback(@(cCompute)d.dat.mCopySeg(d))' '@(varargin)d.mMenuCallback(@(cCompute)d.dat.mPasteSeg(d))'	customization of file menu
ROI hotkeys	{'1', '2', '3'}	selection of region of interest (ROI)

To create the cComputeFatquant.m class file one can use a copy of the template file cCompute_template.m provided in the Dicomflex Git repository. Once the new file is opened in Matlab, modifications and additions can be made. The first task is to customize the properties block of the class by adding entries pVersion_cComputeFatquant and pRoiData. Entry pRoiData is defined as structure-array element storing coordinates of the ROIs, the source data for fitting and the resulting fit parameters. The methods for data access (mPatientName, mSliceLocation and mGetPDFF) and methods defined in the design phase (mGetMagImages, mFitIntensities) are implemented in the methods block.

In general, a key-press event is forwarded from the cControl class to the cCompute method mKeyPress. Colored ROI contours can be selected for modification by pressing a numerical shortcut ('1' to '3'). The remaining contours are greyed out in mKeyPress and will be colored again via mKeyRelease. ROI creation and modification are invoked by selecting and clicking in the image area. A callback for mouse motion (mImgAxisButtonMotion) is set in mImgAxisButtonDown. This method records the cursor coordinates until mImgAxisButtonUp is triggered and the motion callback is released. Old coordinates are merged with the drawn ones by the cCompute method mMergeContours and then displayed. Subsequent fitting of the mean ROI intensities is done by calling mFitIntensities. The fit coefficients are stored in the pRoiData property and available for later calculation of fat fractions (mGetPDFF). Lastly, the UI-update routine is triggered to refresh table entries and graphs.

The process for creating a new dataset is already contained in the framework except for the cComputeFatquant method mInit_oComputeApp that populates all cComputeFatquant objects with application-specific data. In the interest of clean coding, all data are accessed by the defined class methods mPatientName, mSliceLocation and mGetPDFF, avoiding storage of unnecessary data and reducing maintenance. For existing datasets to be loaded, the cComputeFatquant method mUpdatecComputeFatquant can be modified after version changes, but remains untouched for the first version of the Fatquant example.

So far, Fatquant is capable of loading datasets but cannot visualize them on the existing UI. To do so, the mGetImg2Display, mPostPlot, mDrawGraph and mGetTextBoxLines methods in cComputeFatquant are customized. The generic cCompute method mImageUpdate clears the image display of any residual data (contours and image) before a new image is requested from mGetImg2Display to be displayed. A method named mDrawContour is already contained in cCompute and used in mPostPlot to draw all contours stored in the property pRoiData. Text information displaying the current slice number and pImageType is overlaid on the image. The graph area is updated by plotting the data points stored in pRoiData and the fitted curve. The user should then visually inspect the goodness of fit. Application-specific saving of datasets is done in mSaveData. The resulting spreadsheet file is only created if the coefficient of determination (R^2^) lies above an empiric threshold. Otherwise a warning message will be shown.

**Fig 6:**
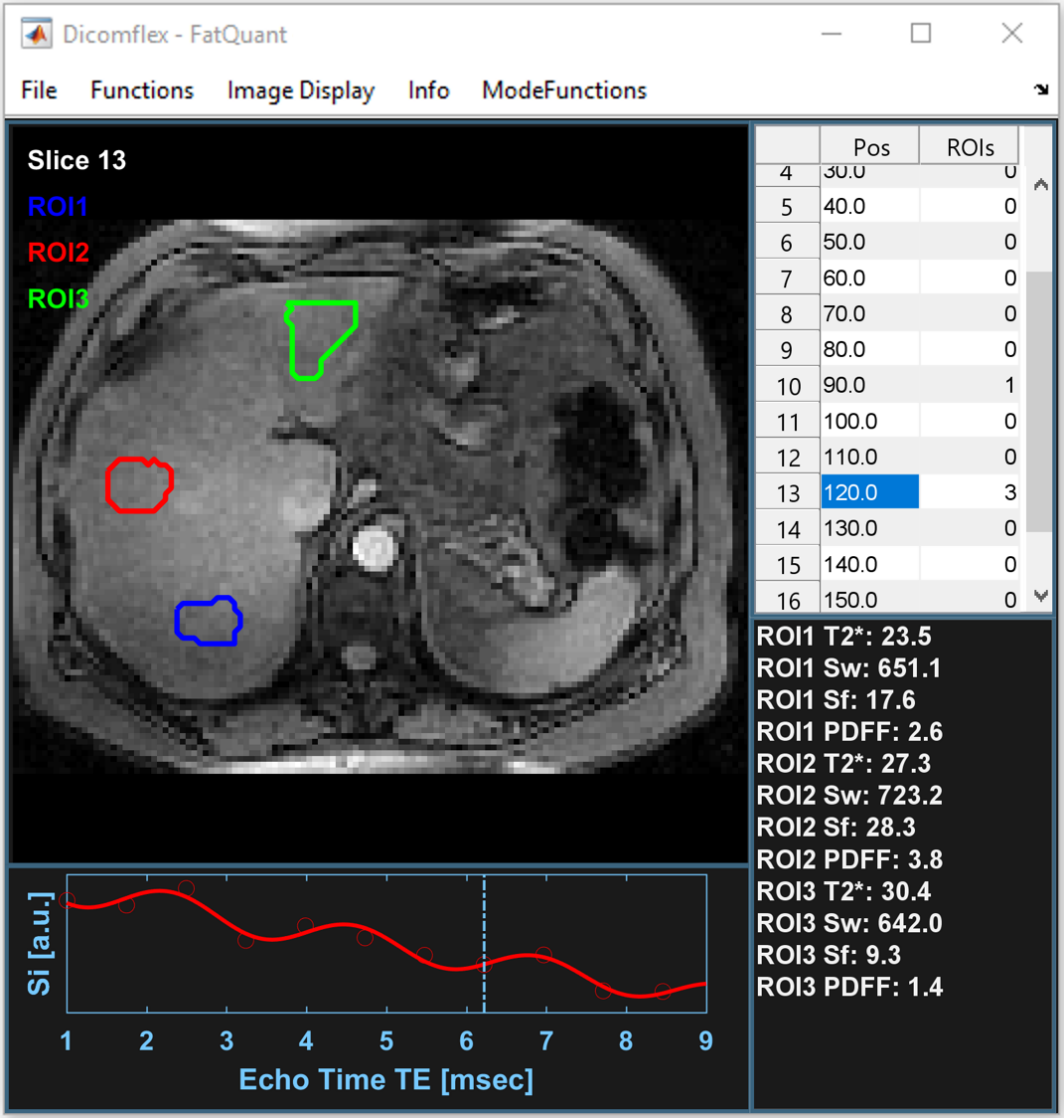
Snapshot of the Fatquant UI. A session file is opened and three ROIs are draw at different positions in the liver. At the bottom left one can see the fit according to ROI 2. At the bottom right, all fit parameters and the resulting PDFF are written.

The Fatquant application was ultimately deployed after all functionalities had been tested within the developer environment as pictured in Fig 6. Fatquant is a frequently used example tool of Dicomflex, which took two days in total to create the first stable executable.

Our preliminary user experience with Dicomflex is based on tools that have exclusively been used in a single medical institution so far. We have replaced the independently developed applications for slice-by-slice analysis of MRI and CT datasets. Initially untrained operators reported a short learning phase and found handling to be intuitive. They also highlighted the benefit and comfort of having a standardized user interface and workflow for different applications. Experienced operators subjectively rated operation of Dicomflex applications to be more fluent than the original tools. As the framework already provides the entire program structure, developers can focus on specific user interactions and processing functions instead.

## Discussion

This work has presented a novel software framework for the analysis of medical image datasets. Dicomflex assists the programmer in creating robust end-user applications with an optimized set of essential UI elements and functions. Development was driven by the systematic and repeated processing needs typically encountered with multislice 2D image data. Another key aspect was the suitability for routine evaluation of clinical study data. Object orientation and explicit handling of versions will increase the applicability and reduce overall software maintenance. The use case described the successful development of a stand-alone application that quantifies fat fractions by fitting a model function to MRI datasets.

Despite the apparent confinement of a predefined framework structure, developers may take advantage of all Matlab features, such as automatic memory management, extensive libraries for numerical and image processing, broad technical references, a large user community and an application compiler. More importantly, DICOM writing is freely available to the Matlab community and does not require additional licenses, unlike, for example, IDL. As a derivative of Matlab, Dicomflex maintains all its benefits. Image processing libraries, such as VIGRA[9] or ImgLib2[10], might be applicable as well. Another example is a complementary approach (ImFEATbox) by the Universities of Tubingen and Stuttgart, Germany, who have assembled Matlab-based functions with reportedly thousands of image features and parameters[11].

Fatquant and other Dicomflex tools have been in routine use over 14 months. Users specifically acknowledged the standardized workflow and user interface for different applications. Main benefits for the developer are the reduced maintenance and ease of creating new Dicomflex tools. Although our framework was developed under Matlab, the concept can be adapted to other programming languages as well. The choice depends on factors like image processing capabilities (libraries and interfaces), ease of programming (memory or automatic data type management), platform independence or ease of creating generic[12] code. Examples include the GNU octave project, an open-source Matlab clone, and the technically similar Python language, which is also open source and has a growing body of functions.

The presented approach is inherently limited to applications for 2D multislice data that meet the predefined workflow. Extensions for 3D image processing and visualization are nevertheless possible. The use case involved MRI analysis in the liver and abdomen, but other regions and tasks are possible as well. Segmentation, computation of parameter maps and ROI-based analyses of any multislice image data are very common, among others, for the assessment of cerebrovascular diseases[20], cardiac function[21] or cancerous tumors[22]. Although these analyses can be performed with fully fledged proprietary software, Dicomflex may be superior for rapid, customized analyses or vendor-independent solutions. The complete framework is available as a Git repository at https://github.com/Stangeroll/Dicomflex and new tools will be released as the development progresses.
